# Direct observation of hematopoietic progenitor chimerism in fetal freemartin cattle

**DOI:** 10.1186/1746-6148-3-29

**Published:** 2007-11-07

**Authors:** Mikael Niku, Tiina Pessa-Morikawa, Juhani Taponen, Antti Iivanainen

**Affiliations:** 1 Department of Basic Veterinary Sciences, Division of Anatomy, University of Helsinki, Helsinki, Finland; 2 Department of Production Animal Medicine, University of Helsinki, Helsinki, Finland

## Abstract

**Background:**

Cattle twins are well known as blood chimeras. However, chimerism in the actual hematopoietic progenitor compartment has not been directly investigated. Here, we analyzed fetal liver of chimeric freemartin cattle by combining a new anti-bovine CD34 antibody and Y-chromosome specific in situ hybridization.

**Results:**

Bull-derived CD34^+ ^cells were detected in the liver of the female sibling (freemartin) at 60 days gestation. The level of bull-derived CD34^+ ^cells was lower in the freemartin than in its male siblings. Bull (Y^+^) and cow hematopoietic cells often occurred in separate clusters. Around clusters of Y^+^CD34^+ ^cells, Y^+^CD34^- ^cells were typically observed. The thymi were also strongly chimeric at 60 days of gestation.

**Conclusion:**

The fetal freemartin liver contains clusters of bull-derived hematopoietic progenitors, suggesting clonal expansion and differentiation. Even the roots of the hematopoietic system in cattle twins are thus strongly chimeric from the early stages of fetal development. However, the hematopoietic seeding of fetal liver apparently started already before the onset of functional vascular anastomosis.

## Background

Cattle twins are well known as blood chimeras [1]. Vascular anastomosis occurs in about 92% of cases, from the 10- to 15-mm crown-rump stage or 30–35 days gestation [2-5]. The blood is thus effectively mixed between the fetuses for most of the 280-day gestation. Postnatally, the twins permanently share composite blood types. This suggests that hematopoietic stem cells (HSCs) are exchanged and successfully engraft in the recipient [6]. However, direct assessment of chimerism in the hematopoietic progenitor compartment has not been possible due to technical limitations.

Donor-derived cells can be readily identified in a cow born as a twin to a bull, as only bull cells contain a Y chromosome. Such chimeric females are usually nonfertile and are called freemartins [7]. We have previously used genomic *in situ *hybridization to analyze the fates of bull-derived cells in freemartin tissues [8]. Recently, we produced an antibody against the bovine sialomucin CD34 for identification of cattle hematopoietic progenitors [9]. Here, we combine these tools to directly observe bull-derived hematopoietic progenitors in the liver of an early fetal freemartin.

## Results and Discussion

**Figure 1:**
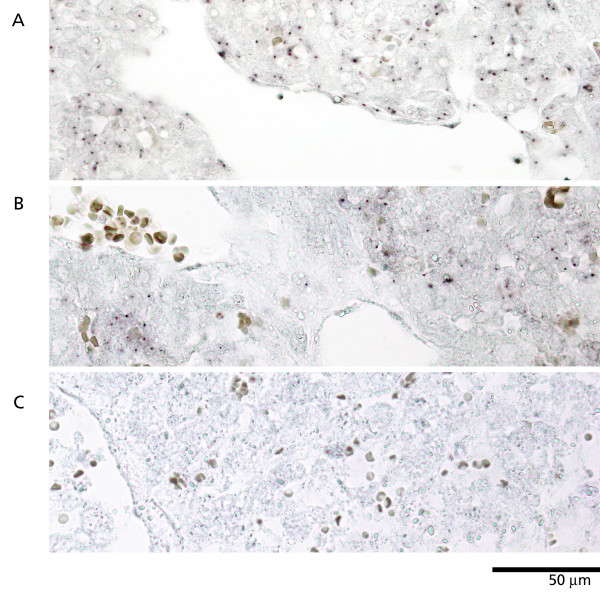
Y-chromosome specific *in situ *hybridization to fetal bovine liver. Dark nuclear spots represent Y chromosomes. A: Chimeric male. B: Chimeric female (freemartin). C: Normal female (from another pregnancy). Erythrocytes were stained with diaminobenzidine.

Fetal liver and thymus were obtained from triplet fetuses in a superovulated cow. The developmental age of the fetuses was 60 days gestation [5]. At this stage of mammalian development, the fetal liver is the major hematopoietic organ [10-13]. The sex of the fetuses was determined by Y-chromosome-specific *in situ *hybridization (Y-ISH) to nonhematopoietic tissues. Two of the fetuses were male (Fig. 1a; note the positive endothelial cells), while one was confirmed as a chimeric female (Fig. 1b).

**Figure 2:**
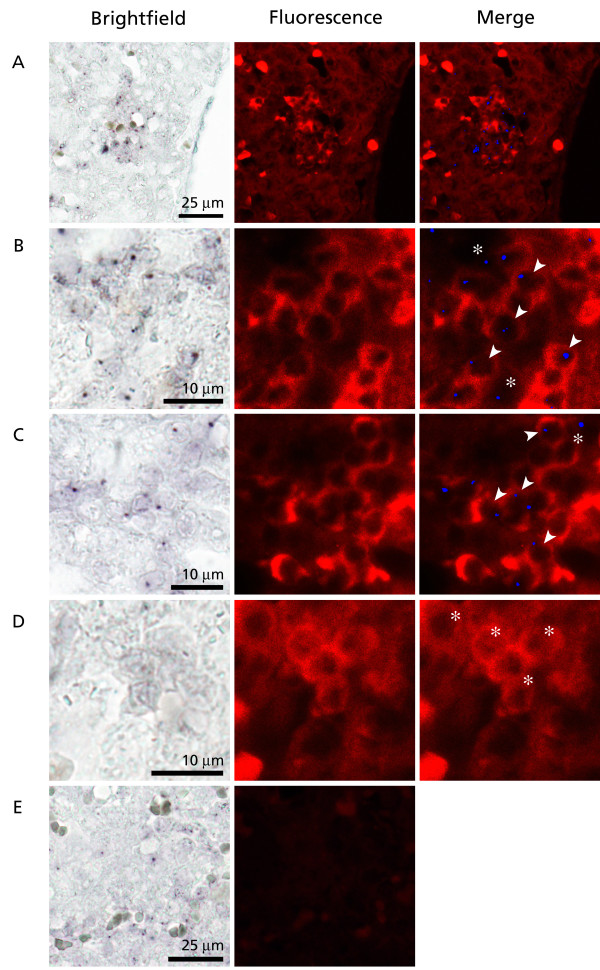
Combined Y-chromosome specific *in situ *hybridization and anti-CD34 immunofluorescence to fetal bovine liver. A-C: Y^+^CD34^+ ^cell clusters in the chimeric female (freemartin). D: A Y^-^CD34^+ ^cell cluster in the chimeric female. E: Negative control without the primary antibody. Arrowheads: Y^+^CD34^+ ^cells. Asterisks: Y^-^CD34^+ ^cells.

The liver of the freemartin fetus was then analyzed by combining Y-ISH and anti-CD34 immunofluorescence (Fig. 2). Livers from normal nonchimeric male and female fetuses were used as positive (Fig. 1a) and negative (Fig. 1c) controls, respectively. Double positive (Y^+^CD34^+^) cells were frequently observed in the freemartin liver, indicating the presence of bull-derived hematopoietic progenitors. Y^+ ^and Y^- ^cells often occurred in separate clusters, suggesting local clonal expansion (Fig. 2a,d). Around Y^+^CD34^+ ^clusters, Y^+^CD34^- ^cells were typically observed (Fig. 2b,c). These probably represent differentiating cells derived from the Y^+ ^progenitors. Alternatively, these may be nonhematopoietic stromal cells.

In the freemartin liver, 22 ± 4.1% of CD34^+ ^cells were Y chromosome positive, while the proportions in the livers of the male siblings were 31 ± 5.4% and 38 ± 6.7% (Table 1). The freemartin liver thus contained significantly less double positive cells (p = 0.004 and p < 0.001, respectively). The difference between the two bull fetuses was not statistically significant. The thymus of the freemartin fetus also contained high numbers of Y^+ ^cells. Here, the differences between animals were less pronounced than in the liver and masked by interlobular variation. There is a strong correlation between levels of bull-derived white blood cells in freemartin calves and their twin brothers ([14], and our unpublished results). The vascular anastomoses in multiple pregnancies enable lymphohematopoietic tissues like the liver, thymus and the bone marrow of each fetus to be populated from a common circulatory pool of progenitor cells. The relatively low level of bull-derived CD34^+ ^cells in the female fetus in the current study is compatible with the seeding of liver beginning prior to the establishment of a functional anastomosis, which normally occurs at around 30 to 35 days of gestation [2-5]. Alternatively, a portion of the immigrant cells could reach the liver directly by migrating through the tissues via a nonvascular route. The interlobular variation in the level of bull-derived cells in the thymus probably reflects corresponding fluctuation in the early circulatory pool of migratory lymphoid progenitors. The interlobular differences are no longer apparent in twin calves and juvenile animals ([14], and our unpublished results) as thymus continuously accepts new lymphoid progenitors.

**Table 1:** Quantification of bull-derived CD34^+ ^cells in the livers of bovine triplet fetuses. Proportions of Y-chromosome positive (Y^+^) cells among liver CD34^+ ^cells in the 60-day fetuses are shown, as detected by in situ hybridization. fm = freemartin, n = number of image fields analyzed.

Fetus	n	CD34^+ ^cells total	% Y^+ ^(mean ± SD)	*P*
fm	7	820	21.8 ± 4.1	<0.01 (bull 1) <0.001 (bull 2)
bull 1	7	518	30.9 ± 5.4	<0.01 (fm) >0.05 (bull 2)
bull 2	5	586	38.4 ± 6.7	<0.001 (fm) >0.05 (bull 1)

We have previously shown that the Y-ISH method applied here is extremely specific and sufficiently sensitive [8]. No false positives were detected in more than 1000 samples of various tissues from normal females (not shown and Fig. 1c). A proportion of bull-derived cells are not detected, as the Y chromosome may be excluded from the section. In normal bull tissues, generally 40–70% of cells are labelled depending on the cell type (here, see Fig. 1a).

CD34 is a transmembrane glycoprotein commonly used as a marker for enrichment of human hematopoietic stem cells [15]. While the CD34^+^fraction is heterogenous and contains also some committed precursors [16], CD34 remains as one of the best known markers for primitive hematopoietic cells [17]. Recently, bovine CD34^+ ^cells were shown to be enriched for hematopoietic progenitors as measured by BFU-E (burst-forming unit – erythroid) and CFU-GM (colony-forming unit – granulocyte-monocyte) readouts [18]. The polyclonal antibody used in our study specifically recognizes bovine CD34 [9]. In addition, it labels erythroid cells. These were excluded from the analysis by pseudoperoxidase staining, as described in Methods.

## Conclusion

This is the first study to directly demonstrate bull-derived hematopoietic progenitors in fetal freemartin cattle. The fetal liver contains clusters of bull-derived cells, suggesting clonal expansion and differentiation. The lower level of bull-derived hematopoietic progenitors in the freemartin vs. her bull siblings suggest that the seeding of fetal liver starts already before the onset of vascular anastomosis. The level of chimerism in the circulating migratory hematopoietic progenitors thus only partially determines the level of donor-derived fetal hematopoiesis. Despite this, the freemartin hematopoietic system is strongly chimeric already from early stages of fetal development.

## Methods

### Fetal tissues

Fetuses were obtained at an abattoir from a superovulated cow. Superovulatory treatment was initiated 11 days after estrus. The cow was administered intramuscularly (i.m.) total of 360 mg of NIH-FSH-P1 (Folltropin^R^-V, Bioniche, Ireland) divided in decreasing doses at 12 h intervals over four days. Luteolysis was induced with an i.m. injection of 0.15 mg of dexcloprostenol (Genestran^R^, 0.075 mg/ml, Vetcare, Salo, Finland) 60 h after the initiation of the superovulatory treatment. The cow was artificially inseminated 48 and 60 h after the dexcloprostenol administration.

Animal experiments were approved by the local animal welfare authorities.

### Combined in situ hybridization and immunofluorescence

Combined Y-chromosome specific in situ hybridization and CD34 immunostaining were performed essentially as described previously [8].

Here, erythroid cells were first stained by their pseudoperoxidase activity using tyramide amplification and the diaminobenzidine (DAB) substrate (Vector Laboratories), as described previously [9]. Genomic in situ hybridization was then performed, using microwave heating, protease treatment, and a Y-chromosome specific oligonucleotide probe carrying a digoxigenin label. The hybridized probes were detected by alkaline phosphatase-conjugated anti-DIG-F_ab _fragments (Roche) and visualized using the NBT/BCIP chromogen (Roche). CD34 was then detected by immunofluorescence. The sections were subjected to heat-induced antigen retrieval and a protease treatment, incubated overnight in the polyclonal anti-bovine CD34 antibody, and then incubated in Alexa546-conjugated anti-rabbit Ig antibody (Invitrogen). Finally, autofluorescence was quenched by Sudan Black B (Merck).

The sections were examined and photographed using a Leica DM4000 epifluorescence microscope equipped with an Olympus DP70 camera. Merged images were prepared in Adobe Photoshop.

### Statistics

Y-chromosome positive and negative CD34^+ ^cells in fetal livers were counted in merged images of double-stained tissue sections, using the Cell^P image analysis software (Olympus). More than 500 CD34^+ ^cells were counted in each animal. Statistical significance was evaluated using independent samples *t *test.

Y-chromosome positive cells were also counted in fetal thymi. Numbers of Y-chromosome negative cells were estimated based on cell densities in hematoxylin-eosin stained serial sections of the same tissues. In total, more than 2000 thymic cells per animal were included in the analysis.

## Abreviations

BCIP, 5-bromo-4-chloro-3-indolyl phosphate; CD, cluster of differentiation; DIG, digoxigenin; FSH, follicle stimulating hormone; NBT, nitro blue tetrazolium; SD, standard deviation

## Authors' contributions

MN designed and carried out the experiments, participated in the generation of the anti-CD34 antibody and drafted the manuscript. TPM participated in the generation of the anti-CD34 antibody. JT designed and carried out the superovulation procedures. AI participated in the design and coordination of all experiments and in drafting of the manuscript. All authors read and approved the final manuscript.
